# Lessons Learned from the Rapid Implementation of Telehealth Group Psychotherapy at a Safety-Net Health System in the U.S

**DOI:** 10.3390/bs15020154

**Published:** 2025-01-31

**Authors:** Astrea Greig, Emily Benedetto, Irina Livitz, Hsiang Huang

**Affiliations:** 1 Department of Psychiatry, Cambridge Health Alliance, Cambridge, MA 02139, USA; hhuang@challiance.org; 2 Department of Primary Care, Cambridge Health Alliance, Cambridge, MA 02139, USA; ebenedetto@challiance.org; 3 Boston Veterans Affairs Health System, Boston, MA 02130, USA; irina.livitz@va.gov

**Keywords:** group psychotherapy, telehealth, safety-net health system, implementation

## Abstract

There is inadequate availability and access to behavioral health services to meet demand, and this issue amplified during the pandemic, creating a mental health crisis. Group therapy is an effective way to meet this need. The rapid implementation of telehealth group psychotherapy as part of a Primary Care Behavioral Health Integration program in a U.S. safety-net health care setting is described. Implementation lessons are summarized as barriers or facilitators, using thematic analysis of qualitative data from meeting notes. Major facilitators identified include having key staff serve as technology champions, dedicated administrative leadership to operationalize workflows, and communication and collaboration across teams and layers of infrastructure. Major barriers include uncertainty about operational workflows and technological challenges. While group visit volume initially waned, it began to rebound and quantitative analysis of demographic data shows that important underserved populations were reached. Frequent communication, collaboration, and adaptation among teams are critical elements for improving the likelihood of successful telehealth group therapy. It is feasible to expeditiously implement telehealth group psychotherapy in safety-net health care systems with limited resources.

## 1. Introduction

The need for behavioral health services exceeds the availability of behavioral health providers (Health Resources and Services Administration, 2015). Group psychotherapy is an effective and efficient answer to the growing behavioral health treatment demands and the shortage of behavioral health clinicians. This modality is as effective as individual psychotherapy in treating a wide breadth of mental health concerns but can reach more patients than individual therapy (Whittingham et al., 2023). Group psychotherapy has also been shown to specifically meet the needs of underserved populations who experience even less access to mental health services than the general population (Hsu et al., 2020; Whittingham et al., 2023). This is particularly important in safety-net public health settings which serve people who are uninsured, underinsured, or have public insurance, and have multiple social and health needs (Institute of Medicine, 2000). 

The issue of access to mental health care was further amplified during the COVID-19 pandemic, fueling a mental health crisis where many individuals did not have access to behavioral health services but reported a need for it (Pasquini & Keeter, 2022). The pandemic compelled health care systems to rapidly transition to telehealth, including behavioral health services. The literature on telehealth group psychotherapy (TGP) has been limited but growing, and a recent systematic review of early studies of TGP has shown promising evidence (Banbury et al., 2018). Notably, both qualitative and quantitative measures have shown that telehealth group psychotherapy produces patient outcomes similar to in-person group psychotherapy (Banbury et al., 2018; Khatri et al., 2014). TGP, particularly telehealth group cognitive behavioral therapy, can be delivered with the same fidelity as in-person group psychotherapy (Khatri et al., 2014). There is also evidence that TGP is efficacious for multiple mental health conditions such as depression, anxiety, anger, post-traumatic stress disorder, borderline personality disorder, and chronic and acute health conditions (Khatri et al., 2014; Melton et al., 2017; Moreland et al., 2010; Zalewski et al., 2021). 

TGP has advantages such as reducing logistical barriers in accessing care, including time, travel, and distance constraints (Melton et al., 2017). Given these benefits, TGP has been increasingly used with patients living in rural areas or those who live far from health care centers (Chen et al., 2021; Melton et al., 2017). Participants of TGP report that these reduced barriers are appreciated (Banbury et al., 2018). Compared to in-person groups, TGP may also provide a sense of anonymity that helps decrease psychological barriers to accessing care such as shame and/or stigma (Borghouts et al., 2021; Houston et al., 2002). Interestingly, in a recent study examining office-based addiction treatment, both patients and medical providers generally preferred TGP visits compared to individual telehealth visits (Sokol et al., 2021).

Concerns raised about TGP include the possibility of promoting feelings of disconnectedness or isolation (Zalewski et al., 2021). Difficulties with navigating telehealth technology, such as logging onto visits and/or experiencing connectivity issues, are also commonly reported issues which may contribute to a negative experience (Banbury et al., 2018). TGP generally produces more favorable symptom outcomes and is more acceptable to patients when using a live video format as compared to a phone only or text format (Gentry et al., 2019). Despite these concerns, attendees report experiencing social connection and bonding with others in TGP and generally view these groups positively. As such, TGP can still benefit patients with limited technology literacy (Banbury et al., 2018).

TGP can address health disparities in access to health care among vulnerable populations, which were exacerbated during the COVID-19 pandemic. Black and Latinx individuals were disproportionately hospitalized due to COVID-19 (Hsu et al., 2020; Smati et al., 2021) and an integrated behavioral health program rapidly developed for patients with COVID-19 found a significant proportion of Spanish-speaking patients with behavioral health and social needs (Benedetto et al., 2021). When state-level policy interventions were enacted to improve telehealth availability, Black residents and those with Medicaid still had difficulty accessing behavioral health care (McBain et al., 2023). Preliminary work shows that TGP has the potential to improve treatment access to vulnerable and underserved populations, who most often obtain treatment in safety-net public health settings, including those with high clinical risk (Childs et al., 2020; Puspitasari et al., 2021) and complex social needs (McInnes et al., 2014).

Based on this early evidence, there is a need for better understanding the barriers and facilitators for TGP implementation in varied behavioral health settings, particularly in safety-net health care settings, in order to help fill the implementation “gap” (Proctor et al., 2009). This paper aims to identify and describe barriers and facilitators of the rapid implementation of TGP in a safety net health setting in the U.S. during the COVID-19 pandemic in order to help inform the implementation of TGP in other organizations seeking to lessen disparities in behavioral health care access.

## 2. Materials and Methods

### 2.1. Setting and Patient Population

The organization is an urban multisite safety-net community health system with two hospitals and 12 primary care clinics in northeastern U.S. Within the organization, the Primary Care Behavioral Health Integration (PCBHI) program provides systematic behavioral health (BH) screening, follow-up, consultation and treatment, and BH services provided by integrated therapists, paraprofessional mental health care partners (MHCP), and consult psychiatrists, based on a stepped model of care (Joseph et al., 2017). The team sits between the primary care and psychiatry departments. The patient population is diverse and underserved, a quarter of whom speak languages other than English, and approximately 65% are either publicly insured or uninsured. 

### 2.2. Telehealth Visits

Prior to the COVID-19 pandemic, the organization did not offer TGP. All of the organization’s and all of PCBHI’s in-person groups were paused at the beginning of the pandemic due to health safety concerns. Workflows were quickly developed for starting individual BH telehealth visits which went through multiple “Plan, Do, Study, Act” cycles to quickly identify and troubleshoot barriers (Peynetti Velázquez et al., 2020). PCBHI’s TGP service was initiated afterwards using these new workflows for individual telehealth visits and preexisting referral processes for in-person group psychotherapy visits. At the start, Google Meet was approved by the organization as a HIPAA compliant platform for all telehealth visits, where patients can access visits by video or audio using a computer, tablet, or smartphone. The entire organization then moved individual telehealth visits to a different platform and TGP remained on the original platform as the newer platform could not accommodate group visits. 

### 2.3. Stakeholders

**Figure 1:**
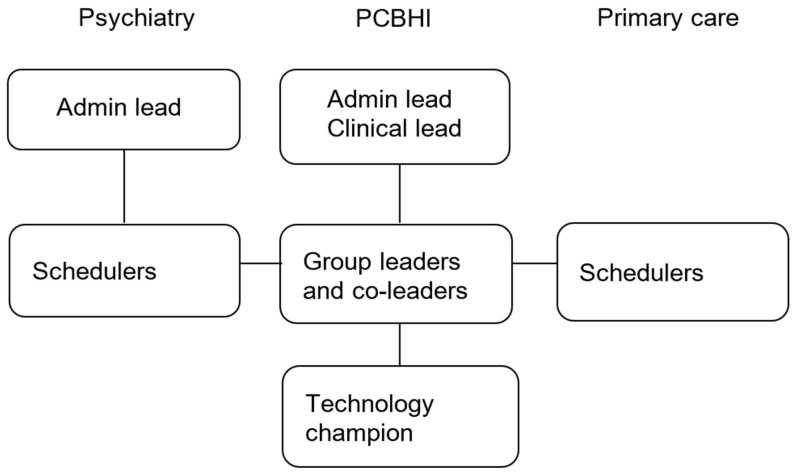
Group implementation roles and relationships by department.

The rapid implementation of TGP required participation from multiple key stakeholders in clinical and administrative roles across departments, including a clinical and an administrative lead from the PCBHI team to direct the creation of TGP offerings and development of workflows; an administrative lead in the psychiatry department; visit schedulers based in both psychiatry and primary care; group leaders and co-leaders; and technology champions. MHCPs most often served as technology champions and/or as group co-leaders. Figure 1 illustrates these roles.

### 2.4. Implementation Plan

The Replicating Effective Programs (REPs) implementation framework was used as a guide for ongoing learning and adaptation during this rapid TGP implementation. The REP is a conceptual implementation framework that is focused on implementing evidence-based practices into community-based health settings that often have limited resources and diverse patients with high rates of medical comorbidity (Kilbourne et al., 2007). The urgent need for telehealth services at the start of the 2020 pandemic necessitated a framework that could be implemented quickly and flexibly. Given these challenges, the REP implementation framework helped ensure fidelity to evidence-based practices while also providing flexibility (Kilbourne et al., 2007). Our implementation plan focused on identifying barriers and facilitators at each phase of implementation and making ongoing adjustments to workflows to improve the quality of care. REP typical stages are shown alongside our implementation plan in Table 1.

### 2.5. Data Collection

Qualitative data were gathered from the groups’ workgroup meeting minutes, which had PCBHI, psychiatry, and/or primary care staff attendees, and any PCBHI daily huddle minutes that included any reference to TGP. In these meetings, stakeholders were encouraged to share both clinical and operational issues. Team huddles occurred with daily frequency during the height of the pandemic and later decreased in frequency, ultimately stopped as the need for increased communication reduced. All of these meetings occurred during 2020. Quantitative data were gathered from the electronic medical record including group visit volume, unique group patient volume, and clinic locations. Demographic data were also compared to the year of visit. Specifically, 2019, before the COVID-19 pandemic, and use of TGP was compared with data from 2020 and 2021 where TGP was used.

### 2.6. Data Analysis

Qualitative data were analyzed using thematic analysis, also known as reflexive thematic analysis (Braun & Clarke, 2006). This analysis used inductive coding and was completed in an iterative manner by the first and second authors. The thematic analysis was conducted using a constructionist approach due to data being collected from meeting notes. Given that meeting notes most often did not consist of direct quotes from staff, the data available are in the context or perspective of the note writer, which therefore makes a constructionist approach most fitting. Quantitative data were analyzed using Chi Square analysis to determine if there are significant associations and then Cramer’s V coefficient was used to measure effect size.

### 2.7. Implementation

Table 2 outlines the four phases of implementation over the course of 16 months, April 2020 to August 2021, and includes groups that were launched and key implementation activities that occurred.

#### 2.7.1. Implementation Phase 1

Multiple meetings with clinical and administrative staff across the PCBHI team, psychiatry, and primary care departments were convened quickly. These initial meetings aimed to identify and prioritize clinical needs and barriers, build TGP workflows, and troubleshoot difficulties. Later, community workgroup meetings started which discussed clinical and administrative tasks that would improve TGP service delivery. Coinciding with the pre-conditions phase of the REP framework, these stakeholders also helped to draft a package of workflows for staff and patients alike to implement TGP. Scheduling staff shortages in primary care required a pivot to temporarily use administrative staff from the psychiatry department. Administrative staff also called patients to provide verbal instruction on how to join TGP in the patient’s language of care. However, our implementation piloted the first telehealth groups in this phase. Therefore, Phase 1 of our implementation combined the REP pre-conditions and pre-implementation phases due to time urgency. To meet relevant clinical needs of the patient population during the pandemic, two telehealth groups were started, a Spanish-speaking anxiety group and a group addressing COVID-related bereavement. Other staff were asked to hold off on starting or restarting prior groups during this initial phase.

#### 2.7.2. Implementation Phase 2

We further refined our package of workflows for both patients and group leaders and also piloted further groups. This aligns with the REP pre-implementation step of piloting the test package. The TGP patient instructions previously provided by phone were now sent electronically. All materials were translated to the organization’s main non-English languages of service (Spanish, Portuguese, and Haitian Creole) and posted on the organization’s website. Four new telehealth groups were launched. These groups were either new or re-launched groups, based on patient population need and/or therapist interest. Meanwhile, PCBHI program leadership established a checklist to guide the process of starting TGP. 

#### 2.7.3. Implementation Phase 3

In this phase we invited more stakeholders across the organization to participate in the community workgroup. The focus shifted to optimizing telehealth group visits across the organization. Building on an internal listing of TGP, the process for advertising new and available groups was better established, with regularly occurring announcements in huddles and emails. Additionally, staff were encouraged to start further TGP and given increased administrative time in order to do so. Three more TGP groups were launched.

#### 2.7.4. Implementation Phase 4

In this phase, we needed to re-organize scheduling workflows due to ongoing staffing shortages. This work was absorbed into standard tasks within the scheduling team as TGP became a more familiar service. Group visits expanded from focusing solely on psychotherapy to also including support groups run by MHCPs as well as medical group visits run by primary care staff. This coincides with the REP maintenance and evolution stage which includes re-customizing the package as needed, organizational changes, and dissemination of the package to a broader population.

## 3. Results

During a 16-month time period, 12 telehealth groups were initiated (see Table 2) in PCBHI. Two groups ended during this time period, one due to low show rate and the other due to the provider’s preference to switch to a different group. Two groups were conducted in Spanish and the rest were in English. 

**Figure 2:**
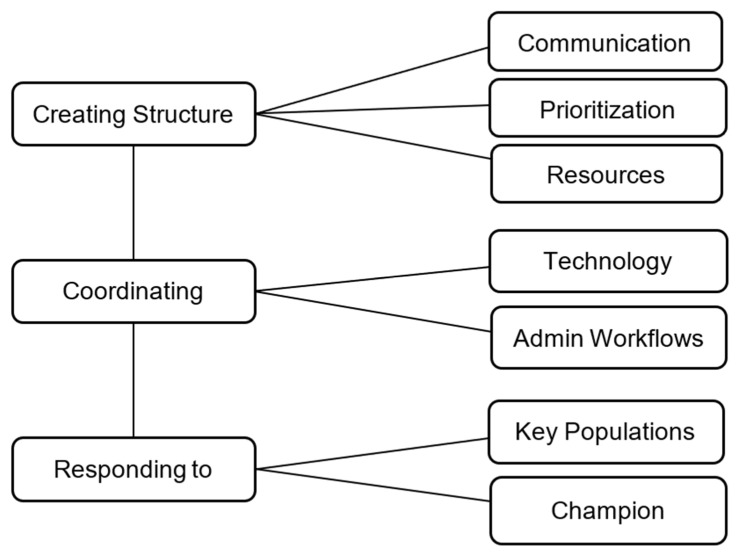
Thematic analysis themes and sub-themes.

Regarding our thematic analysis, three main themes were identified using data from the meeting notes (n = 16). The themes are displayed with their sub-themes as a thematic map in Figure 2.

The first theme, Creating Structure, illustrates how the implementation of TGP involved the need to first focus on Prioritization, Communication, and Resources, which are sub-themes, as a foundation before further work could commence. In order to start TGP implementation, much time was spent discerning when TGP could start in the context of uncertain and/or new resources while needing to increase communication with various stakeholders to ensure success. In the following example quote from the data, we see a need to ensure there are adequate and open lines of communication in order to create the structure by which the rest of the work could follow: “Create a shared folder for workflows and trainings which can be adapted and distributed…” Additionally, it highlights a desire for collaboration across teams and/or departments. Having access to lines of communication to share information as referenced in the following quote, “consider options for peer support of group leaders such as through a list serv”, seems to be desired by staff. Moreover, a need for resources was expressed in the following: “Requesting staff support—central group coordinator and each group has its own support.” This reveals the need for adequate resources in order to make TGP feasible to start. Both sub-themes of Communication and Resources enabled the organization to triage when TGP could occur which describes the sub-theme Prioritization. Prioritization is shown in the following: “Discussion underway about PCBHI’s role in post-acute COVID care, resuming BH screening in televisit process, and Groups,” and “PCBHI also in process of prioritizing new groups related to anxiety, trauma, grief, post-acute COVID, and support for patients from [C]entral [A]merica who seem to be most impacted.”

The second theme, Coordinating Workflows, consists of sub-themes Technology and Admin Workflows. The sub-theme Technology encompasses how new workflows needed to be created for the use of a telehealth platform for both clinical and administrative staff. Concerns arose about the telehealth technology adequately serving patients, as shown here “unsure whether the MyChart/EPIC process is applicable to groups—not yet”, and here “Patient communications, availability in Spanish[?]” The sub-theme Admin Workflows reveals how administrative staff, and the need to standardize their tasks, were vital overall to this implementation. This is depicted in the following: “we’re trying to figure out how…where to get resources… staff who can coordinate/schedule group televisits… which require a specific workflow to set up (including introducing pt to technology/app) and then do regular appt/televisit reminders.” This builds upon the first theme, showing the need to identify logistical workflows by which administrative staff and available technology can be utilized to start TGP implementation. 

Finally, the last theme, “Responding to clinical need” further builds upon the two prior themes. With the foundational structure and the intermediary workflows as discussed in the other themes, the organization’s clinical needs can be addressed. The sub-theme Key Populations refers to the safety-net organization’s vulnerable and complex population and a need to ensure this population is adequately served. This is referenced in the following: “Noted barrier for multicultural/linguistic teams, that patients are less willing to seek care, but may be more willing to access phone over video to preserve anonymity.” Moreover, the sub-theme Champion explains the importance of clinical staff being able to receive support from others such as other therapists or a MHCP. The desire to have a champion is demonstrated here, “every group should have a champion and a team”, and here, “Want to involve care partners.” Having this champion support helps towards the goal of responding to the clinical need of the organization’s populations.

Table 3 illustrates the volume of TGP visits and also patient demographics, including insurance type, among the PCBHI team from the years 2019 to 2021 consecutively. 

Demographically, those that attended groups in 2019 were evenly split across gender (females 50%). However, in 2020 and 2021, after TGP was implemented, there were significantly fewer male patients (X2 (2, N = 2668) = 192.46, *p* < 0.001), with a Cramer’s V of 0.27 indicating moderate effect size. Regarding age, prior to 2020 no groups were offered to patients under the age of 18, thus group attendees under 18 increased in 2020 (1%) and further increased in 2021 (9%). As for adults, the 18–39 years old group increased in 2020 (10%) and stayed consistent in 2021, while other age groups, 40–59 years old and those 60 and over, significantly decreased in 2020 and onwards (X2 (4, N =2668) = 70.79, *p* < 0.001), with a Cramer’s V of 0.12 indicating small effect size. As for race and ethnicity, in 2019 the group attendees were mostly white (74%) with other (12%), Latinx (10%), and Black (9%) groups being the next largest groups, respectively. There was a significant decrease in white patients over the next two years (X2 (8, N = 2668) = 154.62, *p* < 0.001), with a Cramer’s V effect size of 0.17 indicating small effect. The proportion of Latinx patients significantly increased from 2020 onwards (X2 (2, N = 2668) = 132.86, *p* < 0.001) with a moderate effect size, Cramer’s V of 0.22. This coincides with the start of group visits being delivered in Spanish. Insurance type also changed over time with the use of private insurances significantly decreasing from 2020 onwards (X2 (2, N = 2668) = 60.71, *p* < 0.001) with a Cramer’s V effect size of 0.15, indicating small effect.

## 4. Discussion

Our implementation of TGP shows that it is feasible to deliver telehealth psychotherapy groups in a safety-net health care system’s PCBHI program. Both group therapy and telehealth availability are increasingly important to meet the growing need for behavioral health services (Gentry et al., 2019; Whittingham et al., 2023), especially so for underserved populations seen in safety-net health care settings (Institute of Medicine, 2000; McBain et al., 2023). Additionally, using a flexible implementation approach such as the REP enabled us to work within the context of an organization with limited resources and a medically complex patient population. 

Our implementation reveals that group attendees with public insurance, such as Medicaid or Medicare, who often have lower income and multiple social needs, were able to effectively access TGP. Persons of color, who often are over-represented in safety-net or community health settings (Institute of Medicine, 2000), were also able to continue and/or start group psychotherapy visits after the implementation of TGP. Spanish-speaking patients grew in attendance, reflecting the start of group visits in demonstrating that this population was successfully reached through TGP. Yet group psychotherapy usage among people over age 40 and men declined after TGP implementation. The decrease in older adults using TGP may reflect known difficulty among this populations’ use of telehealth services (Banbury et al., 2018). The decrease in men using TGP aligns with other recent studies that have also found that men use telehealth less than women (Datta et al., 2022). Perhaps this reflects apprehension among men about utilizing a new type of behavioral health service or a sentiment that mental health needs can be put on hold or managed by other means during a pandemic.

This rapid initial implementation of TGP revealed numerous barriers yet also multiple facilitators. Regarding the barriers of this TGP implementation, staff struggled with telehealth technology used for TGP visits. This was especially a concern when working with non-English speaking patients who likely found it difficult to navigate features only offered in English. This could exacerbate existing pandemic disparities for patients who do not speak English as their main language (Flores et al., 2021). Furthermore, it is cumbersome when telehealth platforms are not embedded into or compatible with an organization’s electronic health record (EHR) and/or when different platforms are used for group and individual telehealth visits. Additionally, the lack of enough administrative support or uncertainty of where to find administrative support likely delayed the development and refinement of TGP workflows and the expansion of TGP offerings. As such, staff may have experienced increased workload burdens causing TGP visits to be viewed as arduous. 

As for facilitators, close and frequent communication between all staff and stakeholders was imperative. The established workgroup helped to increase the discussion of starting groups in primary care and across teams in the psychiatry department as the meetings created a space to share increased cross-departmental knowledge-sharing and interest regarding group visits. Improved communication then ensures adequate administrative workflows are developed. Consistent with the REP framework, TGP workflows benefited from being assessed and updated as needed to troubleshoot access issues for patients and staff. Similarly to other studies examined in a recent systematic review, the implementation of telehealth is often initially challenging but later improves over time as workflows improve and staff become more familiar with technology (Banbury et al., 2018). Technology champions, both patient-facing and staff-facing, were a vital facilitator. Clinical staff asked for such champions which revealed their value in reducing workload burden to provide TGP. Likewise, it is recommended that non-clinical administrative staff serve as champions for telehealth medical group visits (Sokol et al., 2021). 

A major limitation to our implementation of TGP was our inability to further plan the roll-out due to the urgency of the 2020 COVID-19 pandemic and the need to meet patient needs quickly. Relatedly, our qualitative analyses examined meeting minute notes as there was no time to create formal surveys of staff and/or patient experiences and this limits our ability to capture their perspectives in this implementation. The quantitative analyses of demographic data over time revealed low effect sizes showing that those outcomes should be taken with caution. To build upon this work, future research should focus on further studies of the barriers and facilitators for TGP for patients and staff alike. This could include formal surveys of both staff and patients regarding their experiences of TGP implementation. Also, further examination of group attendees’ demographic data over a longer time period may help clarify the gender and age disparities in TGP usage. 

## 5. Conclusions

It is crucial that TGP be a vehicle and not an impediment to accessing behavioral health care, especially in safety-net settings. To reduce disparities in accessing TGP, safety-net public health care systems would benefit from an easy to use telehealth platform that integrates with commonly used EHR systems, can support group visits in multiple languages, and can support group psychotherapy visits. Yet even with technology barriers, when health care systems focus on adequate support, communication, and leadership, TGP in a safety net setting is a feasible and effective mode of providing mental health care.

## Figures and Tables

**Table 1:** REP stages and corresponding TGP implementation phases.

REP Stage	REP Interventions	Implementation Phase	Implementation Plan
Preconditions	Identify need Identify effective interventions Identify barriers Draft package	1 Spring/ Summer 2020	Identify stakeholders Leadership meetings Develop scheduling and privacy workflows Start workgroup meetings Initiate TGP in a stepwise manner, start with 2 groups
Pre Implementation	Community working group Pilot test package Orientation	2 Summer/ Fall 2020	Continue meetings Share workflows Identify and troubleshoot major barriers from initial groups Initiate more telehealth groups if feasible
Implementation	Training Technical assistance Evaluation Ongoing Support Feedback and refinement	3 Winter/ Spring 2021	Identify and troubleshoot ongoing barriers Provide support as needed to staff and patients Initiate more telehealth groups
Maintenance and Evolution	Organizational/ financial changes National dissemination Re-customize as needed	4 Spring/ Summer 2021	Ongoing support as needed Encourage more telehealth groups without restriction Assess patient data

**Table 2:** Group implementation phases.

Implementation Phase	PCBHI Telehealth Groups Launched	Implementation Activity
1 Spring/ Summer 2020	Anxiety/stress (Spanish) Pandemic-related bereavement	Leadership meetings Workgroup meetings Scheduling and privacy workflows Trialed and initiated virtual groups
2 Summer/Fall 2020	Stress reduction STAIR trauma COVID grief support (Spanish) New and expecting moms	Optimized instructions and scheduling workflows Checklist for establishing a group BH telegroup listing for PC
3 Winter/Spring 2021	Anxiety CBT Chronic Pain Incredible years (parenting)	Centralized scheduling in PC Standardized consent materials Increased advertising to PC
4 Spring/Summer 2021	Trauma and resiliency Coping with depression Stress management for BIPOC	Discontinued centralized scheduling Initiated provider self-scheduling Trialed Brazilian-Portuguese-speaking support groups Trialed medical group billing codes Increased MHCP-led patient coaching during organization-wide transition to new telehealth platform which could not support TGP

Note. PC = primary care; STAIR = skills training in affective and interpersonal regulation.

**Table 3:** Patient demographics and group visit volume before and during pandemic for PCBHI team.

Demographic	2019 n/% of Visits	2020 n/% of Visits	2021 n/% of Visits
Unique patients	201/100%	170/100%	175/100%
Total visits	1308/100%	648/100%	823/100%
Gender Female Male	649/50% 659/50%	461/72% 182/28% *	603/73% 220/27% *
Age group Under 18 18–39 40–59 60+	0/0% 667/51% 422/32% 219/17%	4/1% 401/61% 140/22% * 98/15% *	84/10% 505/61% 157/19% * 177/9% *
Race Indigenous/NA Black/AA Asian Other White Pacific Islander Unknown	0/0% 120/9% 40/3% 158/12% 971/74% 9/1% 10/1%	0/0% 48/7% 18/3% 178/28% 397/62% * 0/0% 2/10%	0/0% 59/7% 113/14% 151/18% 474/58% * 0/0% 26/3%
Ethnicity Latinx/Hispanic	126/10%	114/18% *	188/23% *
Insurance type Public/Medicaid Private/Commercial Uninsured/Self pay	488/37% 812/62% 8/1%	332/52% 305/47% * 6/1%	444/54% 375/46% * 4/0%

Note. NA = Native American/American Indian. AA = African American. * = *p* < 0.001 compared to 2019.

## Data Availability

The raw data supporting the conclusions of this article will be made available by the corresponding author on request.
